# PITX2 is enriched in highly adipogenic brown preadipocytes

**DOI:** 10.17912/micropub.biology.001570

**Published:** 2025-07-24

**Authors:** Manal Al Dow, Charles Colas, Blandine Secco, Alona Kolnohuz, Nolwenn Samson, Audrey Poirier, Romain Villot, Laurence Belley, Bochra Chadel, Laura Tribouillard, Alyson Dufour, Mathilde Mouchiroud, Yves Gélinas, Mathieu C. Morissette, Mathieu Laplante

**Affiliations:** 1 Centre de recherche de l'Institut universitaire de cardiologie et de pneumologie de Québec (CRIUCPQ); 2 Université Laval

## Abstract

Brown adipose tissue (BAT) serves as a key heat-producing organ required to maintain body temperature and homeothermy in mammals. BAT recruitment and activation deeply impacts metabolic homeostasis in both mice and humans. Despite recent advances, the nature of the factors that functionally characterize brown adipose precursors are still incompletely characterized. Here, we provide a comprehensive transcriptomic analysis of brown preadipocyte cell lines with low or high adipogenic potential. Using this resource, we report the identification of paired-like homeodomain 2 (PITX2) as a transcription factor highly enriched in committed brown preadipocytes.

**Figure 1. PITX2 is a transcription factor highly expressed in committed brown preadipocytes:**
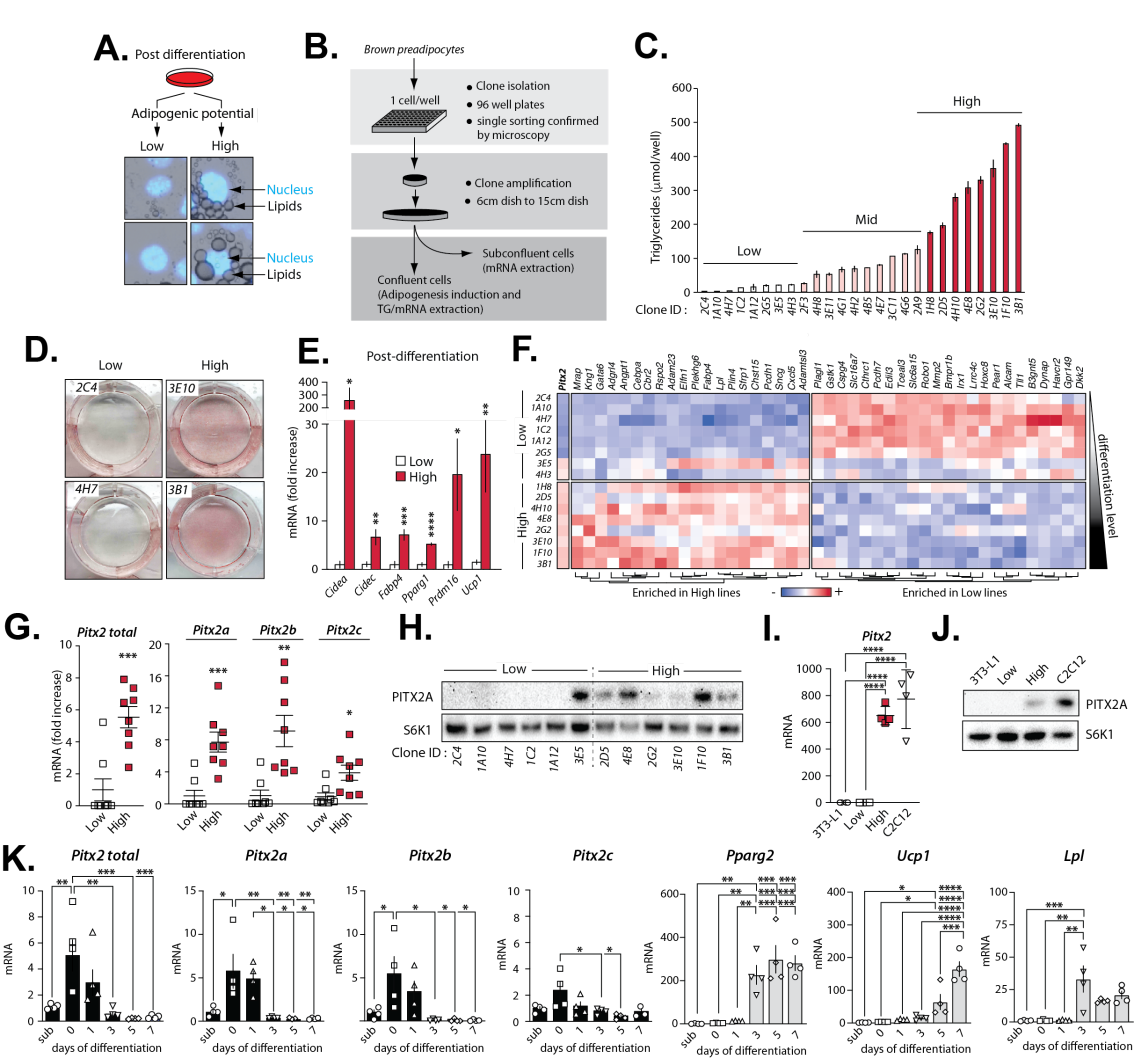
**(A)** Examples of brown preadipocytes showing either low or high adipogenic capacity. Pictures were taken from the same well 5 days following the induction of adipogenic differentiation. Cells were stained with DAPI (blue) to stain the nuclei. **(B)** Schematic presentation of the experimental procedure used to isolate and study clonal lines of brown preadipocytes. **(C)** Triglyceride accumulation measured in 26 clonal lines of brown preadipocytes following the induction of differentiation. Lipid content was measured per well (n=3/cell line) 5 days after the induction of differentiation. **(D)** Oil red O staining of representative Low and High adipogenic lines 5 days following the induction of differentiation. **(E)** Quantitative RT-PCR analyses of adipogenic genes measured in Low (n=8) and High lines (n=7) 5 days after the induction of adipogenesis. **(F)** Heatmap presenting the differential expression profile of several genes between Low (n=8) versus High (n=8) adipogenic lines measured by microarray. The clones are presented in ascending order of their adipogenic capacity. This experiment was performed in pre-confluent cells. **(G)** Quantitative RT-PCR analyses of *Pitx2* isoforms expression in Low (n=8) and High (n=8) adipogenic lines at pre-confluence. **(H)** Western blot analysis of PITX2A protein levels in a subset of Low and High adipogenic clonal lines (n=6/lines). S6K1 was used as a loading control. **(I)** Quantitative RT-PCR analysis of *Pitx2* gene expression in 3T3-L1 preadipocytes, in a Low adipogenic clonal line (4H7), a High adipogenic clonal line (4H10), and C2C12 myoblasts. For all cell lines, a n=4 was used. **(J)** Western blot analysis of PITX2A protein levels in 3T3-L1 preadipocytes, one Low adipogenic clonal line (4H7), one High adipogenic clonal line (4H10), and C2C12 myoblasts. S6K1 was used as a loading control. **(K)** Quantitative RT-PCR analyses of *Pitx2* , *Pitx2a* , *Pitx2b* , *Pitx2c* , *Pparg2* , *Ucp1* , and *Lpl* at sub-confluence (sub) or 0, 1, 3, 5, and 7 days following the induction of adipogenic differentiation (n=4/condition). The parental brown preadipocyte cell line was used in this study. In all panels, data are presented as mean ± SEM. In panels E and G, significance was determined by 2-tailed, unpaired t test. In panels I and K, significance was determined by one-way analysis of variance (ANOVA) with Tukey’s multiple-comparisons test. In all panels, significance is represented as follow *P < 0.05, **P<0.01, ***P<0.001, ****P<0.0001.

## Description

As previously reported, the potential for adipogenesis greatly varies within a culture of white preadipocytes(Green & Meuth, 1974; Gupta *et al* , 2010; Secco *et al* , 2017). Following the induction of adipogenesis *in vitro* , some cells accumulate a large amount of lipids, whereas others are unable to do so. In routine experiments, we found that this phenomenon is also observed when immortalized brown preadipocytes are induced to differentiate into mature fat cells (Fig.1A). Based on these observations, we reasoned that we could take advantage of the adipogenic heterogeneity of brown preadipocytes to identify early genes that characterize brown preadipocytes. Briefly, we performed limiting dilutions of an immortalized brown preadipocyte culture and successfully derived 26 new clonal sub-lines that were next amplified and induced to differentiate (Fig.1B). As presented in Figure 1C and 1D, we found that these clones showed a very divergent capacity for differentiation and lipid accumulation when stimulated with an established adipogenic cocktail. Hereinafter, we refer to these cells as ‘Low’ or ‘High’ adipogenic cell lines. Further supporting the differences in adipogenic capability between the lines, we observed that the expression of terminal and late brown adipogenic markers was elevated in High vs Low adipogenic clonal lines (Fig.1E).

To define the transcriptional signature defining the adipogenic potential of brown preadipocytes, we performed transcriptomic analyses on Low and High adipogenic lines prior to their differentiation, at sub-confluence. As presented in Figure 1F, we observed that the expression profiles of the High adipogenic lines generally clustered together and differed from the Low adipogenic lines. Supporting the validity of our approach, genes previously identified to participate in adipogenesis were identified to be differentially express between the High and Low adipogenic lines. For instance, the expression of *Transcription factor GATA6* ( *Gata6* ) was significantly elevated in preadipocytes with high adipogenic potential (Fig.1F). This transcription factor was recently shown to be expressed in brown fat progenitors and was reported to play roles in brown adipose tissue (BAT) development(Jun *et al* , 2023; Rao *et al* , 2023). Interestingly, we also observed that *CCAAT/enhancer-binding protein alpha* ( *Cebpa* ) and many targets of the nuclear receptor Peroxisome proliferator-activated receptor gamma (PPARG) such as *Lipoprotein lipase* ( *Lpl* ), *Fatty acid binding protein 4* ( *Fabp4* ), and *Perilipin 4* ( *Plin4* ), were significantly elevated in High adipogenic lines. Along with these results, High lines also showed elevated expression of *Secreted frizzled-related protein 1* ( *Sfrp1* ), a secreted Wnt inhibitor previously shown to promote adipogenesis in various contexts(Carson *et al* , 2020; Lagathu *et al* , 2010). Based on these observations, we propose that the transcriptional profiling of Low and High adipogenic cell lines could represent a novel tool to identify and characterize genes playing roles in the early steps of BAT development. The complete gene signature of Low and High adipogenic cell lines is presented in Extended data Table and data is fully accessible through NCBI’s Gene Expression Omnibus (GEO accession number GSE291066).

As presented in Figure 1F, we found the most differentially expressed gene between the Low and High lines was *Paired-like homeodomain 2* ( *Pitx2* ). The expression of *Pitx2* was enriched by 21.3 fold in High lines compared to Low lines. PITX2 is a transcription factor that impacts morphogenesis. In humans, mutations in *PITX2* were linked to Rieger syndrome, an autosomal-dominant human disorder causing anomalies in the development of the teeth, eyes, and abdominal region(Semina *et al* , 1996). Studies in xenopus, chick, and mouse revealed a key role for PITX2 in regulating left-right asymmetry of internal organs(Logan *et al* , 1998; Piedra *et al* , 1998; Ryan *et al* , 1998; St Amand *et al* , 1998; Yoshioka *et al* , 1998). In the last decade, *Pitx2* has emerged as a key regulator of skeletal muscle development as well as the differentiation and cell fate of satellite cells in adult muscle(Hernandez-Torres *et al* , 2017). In mouse, several isoforms of PITX2 have been identified but none of these have ever been reported to participate in brown fat cell adipogenesis. Quantitative RT-PCR analyses confirmed that total *Pitx2* expression was enriched in preadipocytes with high adipogenic potential (Fig.1G, left panel). The same result was observed when using primers designed to amplify specific isoforms ( *Pitx2a* , *Pitx2b* or *Pitx2c* )(Fig.1G, right panels). Western blot analyses using an antibody raised against the amino acids 1-38 of PITX2A confirmed the higher expression of this transcription factor in brown preadipocytes with high adipogenic potential (Fig.1H).

As discussed above, PITX2 contributes to muscle cell development in mice(Hernandez-Torres *et al.* , 2017). This is particularly interesting considering that skeletal muscle and BAT were shown to emerge from a common population of progenitor cells(An *et al* , 2017; Seale *et al* , 2008). To explore how PITX2 expression varies across cell lines of different adipogenic capability and origins, we next measured its levels in white preadipocytes (3T3-L1), in Low and High brown preadipocytes, and in the immortalized myoblast cell line C2C12. We observed that the expression of *Pitx2* was extremely low in 3T3-L1 cells and brown preadipocytes with low adipogenic potential (Fig.1I). Interestingly, *Pitx2* expression was elevated in highly adipogenic brown preadipocytes and in C2C12 cells (Fig.1I). Similar observations were made when PITX2 protein was measured by western blot (Fig.1J). Together, these results confirm that PITX2 is a transcription factor highly expressed in adipogenic brown fat precursors and myoblasts.

In a last set of experiments, we measured the expression of *Pitx2* (total and isoforms) over the course of adipogenesis to profile the expression pattern of this transcription factor during brown fat cell maturation. As presented in Figure 1K, *Pitx2* expression rose in cells transitioning from sub-confluence (sub) to confluence (day 0). This early peak in *Pitx2* expression was observed for all the isoforms measured. A decrease in *Pitx2* levels was next measured from day 1 to day 7 following the induction of adipogenesis. This reduction in expression was associated with the rise in the expression of late markers of adipogenesis including *Pparg2* , *Uncoupling protein 1* ( *Ucp1* ) and *Lpl* (Fig.1K). Overall, these results indicate that *Pitx2* expression associates with the early phases of brown fat cell development *in vitro* .

In conclusion, we report the isolation and profiling of new clonal lines of brown preadipocytes with either low or high adipogenic potential. Using this tool, we identified PITX2 as a transcription factor highly expressed in committed brown preadipocytes. Our work shows that *Pitx2* is expressed in the first phase of brown fat cell development and suggests a possible role for this protein in the early commitment of brown preadipocytes.

**Limitations and future work.**

While our findings show that PITX2 is highly expressed in committed brown preadipocytes, it is important to point out that we did not directly assess its functional involvement in brown fat cell development. Future experiments examining whether overexpressing PITX2 isoforms can restore adipogenesis in Low lines would be valuable in determining if PITX2 directly contributes to adipogenesis. Additionally, testing whether forcing PITX2 expression could influence the expression of brown fat cell markers in white preadipocytes could uncover a broader role for this transcription factor in regulating adipocyte lineage commitment.

## Methods

***Cell culture.*** Immortalized brown preadipocytes were isolated as described(Galmozzi *et al* , 2014) and kindly provided by Dr. Shingo Kajimura. Sub-confluent cells were maintained in Dulbecco’s Modified Eagle Medium (DMEM) with 10% fetal bovine serum (FBS). Brown preadipocytes were induced to differentiate using an adipogenic cocktail containing insulin (5mg/ml), triiodothyronine (T3) (1nM), dexamethasone (2mg/ml) and IBMX (500mM). After 3 days, the media was changed, and cells were maintained in DMEM 10% FBS supplemented with T3 (1nM).

***Lipid extraction and Oil Red-O staining.*** Triglycerides were extracted post-differentiation using the method previously described by Folch(Folch & Lees, 1957). Briefly, cells were scraped in 0.6 ml PBS and transferred to a glass tube. After addition of 3 ml of a chloroform:methanol (2:1), the tubes were vortexed and centrifuged for 10 minutes. The upper aqueous phase was collected and discarded. The remaining lower phase was washed once with water, evaporated, dissolved in 0.1 ml isopropanol, and evaluated for triglycerides content using a standard assay kit (Thermo Scientific, TR22421). For Oil red O staining, cells were fixed with 4% paraformaldehyde (PFA) for 30 minutes at 37°C, washed with PBS andstained for at least 30 minutes with Oil red O. Cells were then washed with PBS and imaged using an Olympus BX60 microscope (Tokyo, Japan).

***RNA extraction and quantitative RT-PCR.*** Total mRNA was isolated from tissues using the RNeasy Mini Kit (Qiagen, 74104). The RNA concentrations were estimated from absorbance at 260 nm. cDNA synthesis was performed using the iScript™ Advanced cDNA Synthesis Kit for RT-qPCR (Bio-Rad, Mississauga, ON, Canada). cDNA was diluted in DNase-free water (1:15) before quantification by real-time PCR. mRNA transcript levels were measured in duplicate samples using a CFX96 or CFX384 touch ^TM^ real-time PCR (Bio-Rad, Mississauga, ON, Canada). Chemical detection of the PCR products was achieved with SYBR Green (Bio-Rad, Mississauga, ON, Canada). Gene expression was corrected for the expression level of reference genes. The following primers were used.

**Table:** 

Gene	Forward primer	Reverse primer
*18S*	*GGCCTGCGGCTTAATTTGAC*	*CATGCCAGAGTCTCGTTCGT*
*Arbp*	*AGAAACTGCTGCCTCACATC*	*CATCACTCAGAATTTCAATGG*
*Cidea*	*AAAGGGACAGAAATGGACAC*	*CCTCAGCAGATTCCTTAACAC*
*Cidec*	*GGCCCGGGTAACCTTCGACCT*	*AGCTGGAGGTGCCAAGCAGC*
*Fabp4*	*GACGACAGGAAGGTGAAGAG*	*ACATTCCACCACCAGCTTGT*
*Lpl*	*GCACTTTCCAGCCAGGATGC*	*GGCCTGGTTGTGTTGCTTGC*
*Pitx2 total*	*CGGCAGAGGACTCATTTCAC*	*CGTTCCCGCTTTCTCCATTTG*
*Pitx2a*	*GAGAGCAGCAGACAGAAAC*	*ATCTTTCTCTAATTGCACGC*
*Pitx2b*	*GGCAGCCGTTGAATGTCTCT*	*GTTGCCGCTTCTTCTTGGAC*
*Pitx2c*	*CTTGGAGCACCGAGCAGC*	*CTGGAAAGTGGCTTCCAG*
*Pparg1*	*TGAAAGAAGCGGTGAACCACTG*	*TGGCATCTCTGTGTCAACCATG*
*Pparg2*	*ACTGCCTATGAGCACTTCAC*	*CAATCGGATGGTTCTTCGGA*
*Prdm16*	*CCAGATGTCAGCCATAGAAAC*	*CACAGTACTTGCACCTGTATGG*
*Ucp1*	*GCAGTGTTCATTGGGCAGCC*	*GGACATCGCACAGCTTGGTAC*

***Western blot.*** Cells were rinsed twice with ice-cold PBS before lysis. Cells were lysed with lysis buffer containing 50 mM HEPES, pH 7.4, 2 mM EDTA, 10 mM sodium pyrophosphate, 10 mM sodium glycerophosphate, 40 mM NaCl, 50 mM NaF, 2 mM sodium orthovanadate, 1% Triton-X 100, and one tablet of EDTA-free protease inhibitors Roche per 25 ml. After lysis, lysates were rotated at 4°C for 10 minutes. The soluble fractions were next isolated by centrifugation for 10 min in a microcentrifuge. Protein levels were then quantified using Bradford reagent. Protein extracts were diluted in sample buffer, denaturated at 95°C for 10 minutes and loaded on precast gels (Life Technologies). Proteins were transferred to PVDF membranes blocked in 5% milk diluted in PBS-Tween and incubated with their primary antibody overnight at 4°C. PITX2A antibody was kindly provided by Dr. Jacques Drouin (Institut de recherches cliniques de Montréal, Université de Montréal) and used at a dilution of 1:4000. p70 S6 kinase was purchased from Cell Signaling Technology (#9202) and diluted 1:1000. Secondary antibodies were purchased for Cell signaling technology.

***Microarray analyses.*** Total RNA was quantified using a NanoDrop Spectrophotometer ND-1000 (NanoDrop Technologies, Inc.) and its integrity was assessed using a 2100 Bioanalyzer (Agilent Technologies). Sense-strand cDNA was synthesized from 100 ng of total RNA, and fragmentation and labeling were performed to produce ss-cDNA with the GeneChip® WT Terminal Labeling Kit according to manufacturer’s instructions (Thermo Fisher Scientific). After fragmentation and labeling, DNA was targeted to be hybridized on Clariom™S HT, Mouse (Thermo Fisher Scientific) and process on the GeneTitan® Instrument (Thermo Fisher Scientific) for Hyb-Wash-Scan automated workflow. Probe set intensities were imported using the oligo R package (v1.70.0)(Carvalho & Irizarry, 2010) and normalized with the Robust Multi-array Analysis (RMA) algorithm(Irizarry *et al* , 2003). Differential gene expression analysis was performed using the limma R package (v3.62.2)(Ritchie *et al* , 2015), and genes with an adjusted p-value < 0.1 were considered differentially expressed. Data is accessible through NCBI’s Gene Expression Omnibus (GEO accession number GSE291066)

***Statistics.*** All the statistical analyses were performed using Prism (version 10.2.3). In all panels, data represent the mean ± SEM. The statistical tests used to determine significance are identified in the figure legends.
